# Genes as Early Responders Regulate Quorum-Sensing and Control Bacterial Cooperation in *Pseudomonas aeruginosa*

**DOI:** 10.1371/journal.pone.0101887

**Published:** 2014-07-09

**Authors:** Kelei Zhao, Yi Li, Bisong Yue, Min Wu

**Affiliations:** 1 Department of Basic Sciences, School of Medicine and Health Sciences, University of North Dakota, Grand Forks, North Dakota, United States of America; 2 Key Laboratory of Bio-resources and Eco-environment, Ministry of Education, College of Life Sciences, Sichuan University, Chengdu, Sichuan, China; National Jewish Health, United States of America

## Abstract

Quorum-sensing (QS) allows bacterial communication to coordinate the production of extracellular products essential for population fitness at higher cell densities. It has been generally accepted that a significant time duration is required to reach appropriate cell density to activate the relevant quiescent genes encoding these costly but beneficial public goods. Which regulatory genes are involved and how these genes control bacterial communication at the early phases are largely un-explored. By determining time-dependent expression of QS-related genes of the opportunistic pathogen *Pseudomonas aerugionsa*, we show that the induction of social cooperation could be critically influenced by environmental factors to optimize the density of population. In particular, small regulatory RNAs (RsmY and RsmZ) serving as early responders, can promote the expression of dependent genes (e.g. *lasR*) to boost the synthesis of intracellular enzymes and coordinate instant cooperative behavior in bacterial cells. These early responders, acting as a rheostat to finely modulate bacterial cooperation, which may be quickly activated under environment threats, but peter off when critical QS dependent genes are fully functional for cooperation. Our findings suggest that RsmY and RsmZ critically control the timing and levels of public goods production, which may have implications in sociomicrobiology and infection control.

## Introduction

Bacteria communicate using quorum-sensing (QS) to coordinate cooperative behaviors, such as production of extracellular factors, which can be used by any community member and are normally beneficial for nourishment, significantly improving population fitness [1]–[4]. From an economic viewpoint, these costly public goods should be strictly controlled and synthesized only when needed [5]. Previous studies have suggested that the expression of exoproduct-encoding genes was much higher at low cell densities under conditions that require these extracellular factors for growth [6]. This indicated that the public goods-induced cooperation might have progression phases and regulated by different genes depending on environmental factors.

Therefore, some overlooked, but important issues are raised here. (*i*) Is production of costly public goods induced at a specific density? (*ii*) Will social cooperation still occur if the conditions do not require the corresponding public goods? (*iii*) Will cooperative behaviors be prematurely activated when an environment threatens the bacterial population at a low cell density? Addressing these questions requires the understanding of environment dependent stabilization of extracellular factors production. However, the genes for regulation of cooperative behaviors at low cell density or early phases are largely unexplored. Defining these early regulators may help understand virulence regulation in response to nutrient variation or environmental threat. Past research also confirmed that the QS system of human pathogen *Pseudomonas aeruginosa* regulates >353 genes (6% of the genome), which primarily function at logarithmic and stationary phases [7]. In particular, the GacS/GacA two-component system, a global regulator of the expression of bacterial products by turning on the transcription of small RNAs, was reported in many gram-negative bacteria including *P. aeruginosa* [8]–[13]. These small RNAs act as antagonists of the unique RNA-binding protein RsmA, which negatively controls the expression of QS-related genes and several extracellular products [14]–[16]. These findings suggested to us a possible molecular mechanism of QS-modulated social cooperation.

Herein, we first divided the growth of *P. aeruginosa* in shaking culture into three phases: low density, quorum density and high density to detect the variation of expression of regulatory and QS-dependent genes. Then the time-dependent expression of QS-related genes was determined in M9****minimal growth medium [1] containing different carbon sources or in conditions with different stresses. As predicted, the small regulatory genes *rsmY* and *rsmZ* were found to act as early responders to regulate the induction of social cooperation to optimize the population density upon environmental stimulation.

## Materials and Methods

### Ethics Statement

The authors declared that this study do not require an ethics statement.

### Bacterial strains and culture conditions

Wild type (WT) *Pseudomonas aeruginosa* PAO1 was a gift from Dr. S. Lory (Harvard Medical School, Boston, MA) [17], [18]. Quorum-sensing defective *P. aeruginosa* strain PAO1-*ΔlasR* was kindly provided by Dr. C. He (University of Chicago, Chicago, IL) [19]. *P. aeruginosa* isogenic mutant strains lacking *gacA*, *rsmY*, *rsmZ*, *rsmYZ* or *rsmA* genes were kindly provided by Dr. Reimmann, Dr. Gabriella, Dr. Pessi, Dr. Humair and Dr. Holden, respectively [8], [14], [15], [20], [21]. Strains were inoculated in LB broth or designated medium with shaking (220****rpm) at 37°C [22].

### Determination of *lasB* expression

To investigate whether there were threshold density at which could induce the cooperation, 10****µl of overnight cultivated WT PAO1 were inoculated into sterile tubes with 2****ml, 4****ml and 6****ml of LB broth medium with shaking at 37°C. The cell density was determined by measuring optical density at 600****nm (OD_600_) once an hour, and activation of cooperation was determined based on the expression of elastase (encoded by *lasB* gene). Subsequently, WT PAO1 were cultured in 4****ml LB broth for 3****h and then manually adjusted to a lower density than the assumed and continued to be cultivated to detect the activation of cooperation. The production of LasB and the final population density were then determined in the presence of *lasR* mutants. Finally, the colony forming units (CFUs) at which the cooperation was induced and final time points were counted.

### Identification of leadership cadre

*P. aeruginosa* PAO1 mutant strains lacking *gacA*, *rsmY*, *rsmZ*, *rsmYZ* or *rsmA* genes were cultured in LB broth medium for 24****h to count the CFU, respectively. Then the total RNAs of different PAO1 mutants were isolated at time points to detect the expression of these genes and *lasR* as well as *lasB* by quantitative RT-PCR using specific primers (Table S1). Subsequently, based on the threshold cell density of WT PAO1 that was determined in shaking cultivation, the growth of WT PAO1 and *lasR* mutant were divided into three phases: low density, quorum density, and high density. Bacterial RNA was isolated at each phase to investigate the variation of the expression levels of QS related genes. The relationship between these genes was analyzed by Spearman’s correlations.

To further elaborate the role of small regulatory RNAs for social cooperation, the growth rates of WT PAO1 and isogenic mutant strains as mentioned above were tested when using adenosine as sole carbon source. The final densities of WT PAO1 cultured in M9****minimal growth medium [1] containing 1% adenosine and 1% BSA which was added after 20****h and 40****h were measured at OD_600_. Subsequently, time-dependent expression of QS related genes were detected in M9****minimal growth medium containing 1% adenosine, 0.5% adenosine+0.5% BSA, 0.5% adenosine+0.5% BSA and the supernatants were removed every 4 hours, 1% adenosine and then 1% BSA was added after 20 hours and 40 hours cultivation.

### Detection of cooperation in stress environments

Mouse alveolar macrophage MH-S cells were obtained from American Type Culture Collection (ATCC CRL-2019) and maintained RPMI/F12 medium (50%∶50%) and 2****mM HEPES buffer. To detect the performance of small regulatory genes *rsmY* and *rsmZ* in different stressful environments, MH-S cells were seeded into 6-well plates (10^9^ cells per well) followed by incubation with 10****µl overnight culture of WT PAO1. The same amount (1.0×10^7^ ****CFU) of WT PAO1 was added into LB broth medium containing 2****µg/ml gentamicin. Total RNAs were isolated at designed time points and the expressions of *rsmY*, *rsmZ* and *lasB* genes were also detected by qRT-PCR.

To determine the effect of anti-virulence drug (5Z)-4-bromo-5-(bromomethylene)-3-butyl-2(5H)-furanone (furanone C-30) on bacteria growth [23], WT PAO1 was first cultured with 50****µM furanone C-30 in LB broth medium and the CFUs were enumerated at designed time phases. Subsequently, furanone C-30-treated PAO1 cells were harvested at the time point that the population began to significantly increase, and immediately diluted to the same cell density (1.0×10^5^ ****CFU/ml) with untreated PAO1 for further cultivation to measure the growth rates. Finally, the expression of *rsmY*, *rsmZ* and *lasB* in furanone C-30-treated PAO1 or WT PAO1 was detected in LB broth, LB broth containing 20****µM 3O-C_12_-HSL and 20****µM C_4_-HSL, and LB broth containing 50****µM furanone C-30.

### Biofilm production

Production of biofilm was detected by crystal violet staining and then quantified at OD_595_ as previously described [24]. Briefly, unattached bacterial cells were removed after culture and the tubes were gently washed with PBS. Biofilms were then stained with 0.2% (wt/vol) crystal violet for 30****min. The excess crystal violet dye was washed out followed by washing the samples with double distilled water. The crystal violet stained biofilm was dissolved by 95% ethanol and measured at OD_595_ for quantification.

### Data analysis and statistics

The results of gene expression were normalized to the levels of housekeeping gene *rpoA* and then calculated using the 2^−*ΔCT*^ method [25]. Data and statistical tests were computed using Graphpad Prism version 5.0 (San Diego, CA). Means were compared using one-way ANOVA, followed by a Tukey-Kramer post hoc test using a 95% confidence interval. Data are shown as means ± standard error means (SEM). Spearman’s correlations were calculated between expression levels of the different genes by Statistical Package for Social Science (SPSS) software (version 18) [26]. All the experiments were performed in triplicate.

## Results

### GacA-RsmY/Z leadership cadre governs the initial expression of the dependent genes

**Figure 1:**
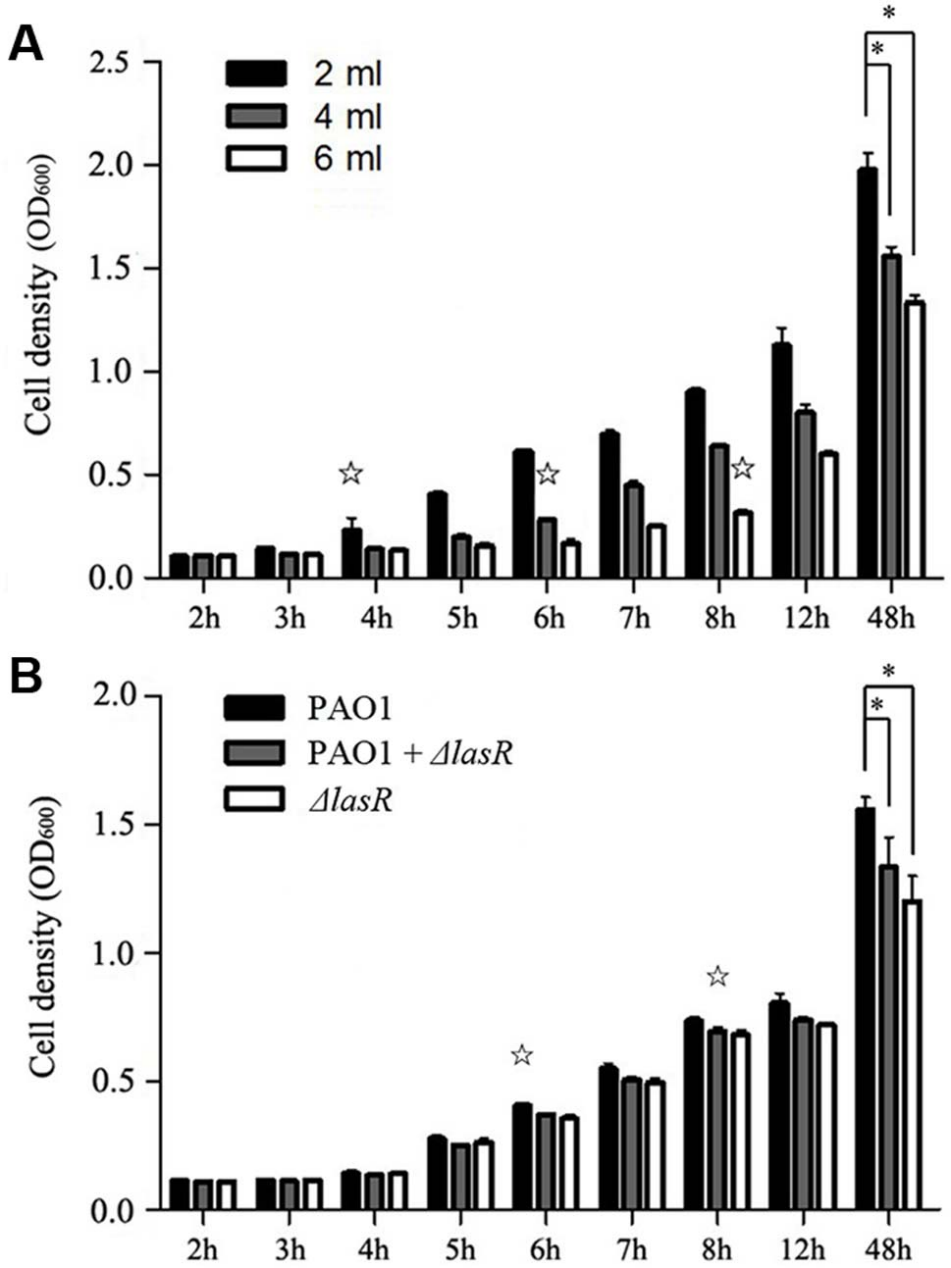
Cooperation activation was delayed due to the presence of *lasR* mutant. (A) The same amounts of wild type (WT) *P. aeruginosa* PAO1 were cultured in different volumes of LB broth medium. (B) 10****µl of overnight cultivated WT PAO1, 5****µl WT PAO1+5****µl PAO1-*ΔlasR*, and 10****µl PAO1-*ΔlasR* strains were independently cultured in the same volume of LB broth medium. Activation of cooperation (☆) was determined by significantly increased expression of *lasB* gene. Data are means ± SEM, and representative of three experiments. *, *P*<0.05, one-way ANOVA (Tukey-Kramer post hoc).

**Figure 2:**
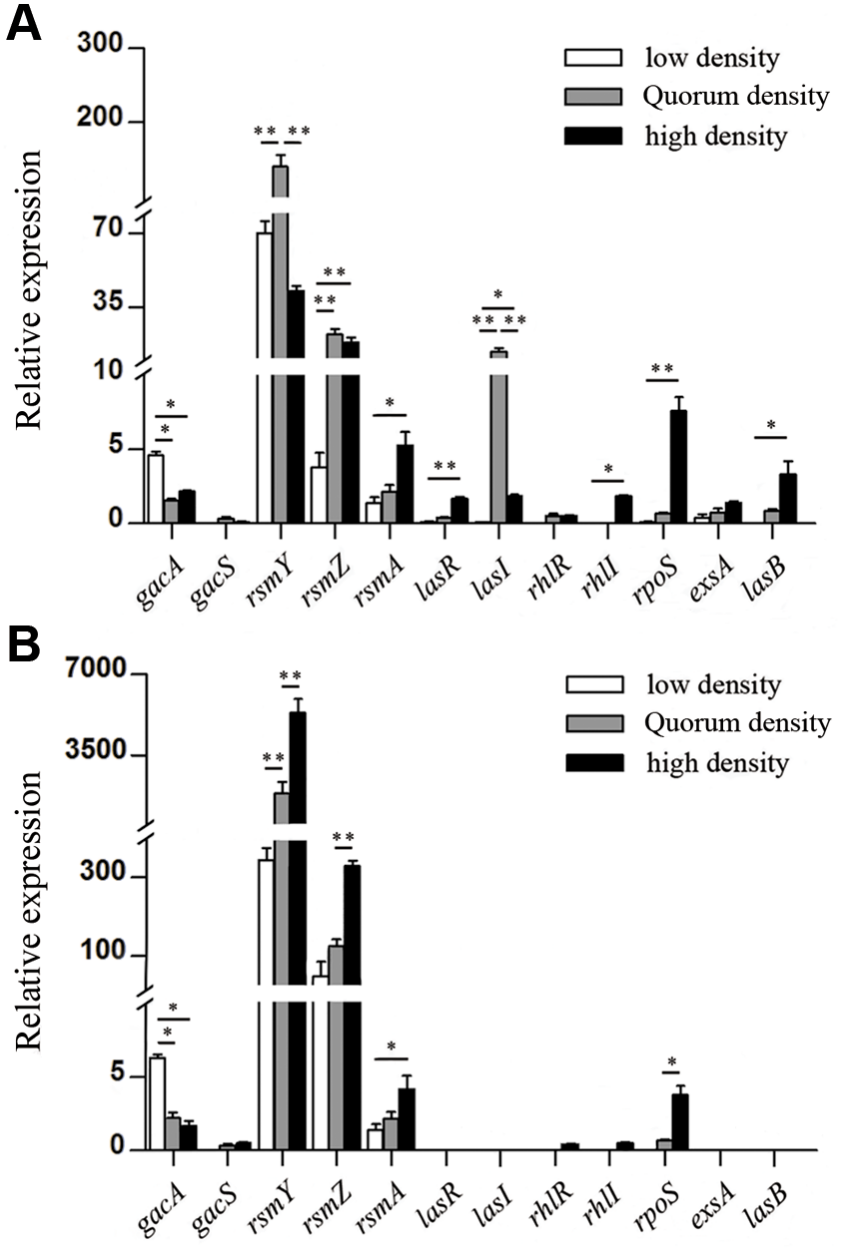
GacA-RsmYZ leadership cascade governs the initial expression of the QS-dependent genes. Gene expression of leadership and QS regulators in WT PAO1 (A) and QS-deficient strain PAO1-*ΔlasR* (B) in low density (OD_600_≈0.12), quorum density (OD_600_≈0.28), and high density (OD_600_≈0.9) phases. Data are means ± standard error means (SEM), and representative of three experiments. *, *P*<0.01; **, *P*<0.001, one-way ANOVA (Tukey-Kramer post hoc).

To explore the regulatory genes responsible for initiating the cooperative activity in quiescent periods, we set out to examine whether production of costly extracellular products (public goods) is induced at a specific population density. The production of public goods at various times was measured by culturing wild type (WT) *P. aeruginosa* PAO1 at different initial densities (ratio of cell numbers to total volume), with increasing expression of *lasB* gene that codes for the typical public good elastase as an indicator for cooperation [1]. The results showed that the expression of *lasB* became detectable at a specific density (∼6.0×10^7^ CFU/ml; Fig. 1A). To detect the variation of expression of regulatory and dependent genes, the growth of WT PAO1 culture was divided into three phases: low density (OD_600_≈0.12), medium density (quorum density, OD_600_≈0.28), and high density (OD_600_≈0.9), which laid the foundation for exploring the initial regulators of cooperative regulation. As shown in Fig. 2A, the global control gene *gacA* was expressed at high levels at low cell density, but was gradually decreased when late responders were activated after 3****h incubation. The expression of two small RNAs *rsmY* and *rsmZ* began to increase at low density and decreased after quorum density, when cooperation was induced (slightly later than *gacA* but ahead of *lasB* expression). Correlation analysis of QS-related genes (Table S2) also confirmed that the global regulatory effect of GacA was dependent on the expression of RsmY and RsmZ [15]. On the contrary, in the absence of the central regulatory QS gene *lasR*, the expression of dependent genes was impaired along with the dramatically increased expression of *rsmY* and *rsmZ* compared with the WT PAO1 (Fig. 2B). Moreover, the growth of *P. aeruginosa* mutants lacking proper regulatory genes was significantly impaired in LB medium (Fig. S1), and these mutants manifested decreased expression of *lasR* and *lasB* and confirmed the hierarchical relationship of these regulatory genes (Fig. S2). Therefore, the GacA-RsmYZ leadership cascade governs the performance of dependent genes, and this dynamic is typically sustained until cooperation is induced.

### Leadership-regulated cooperation was depended on environmental factors

**Figure 3:**
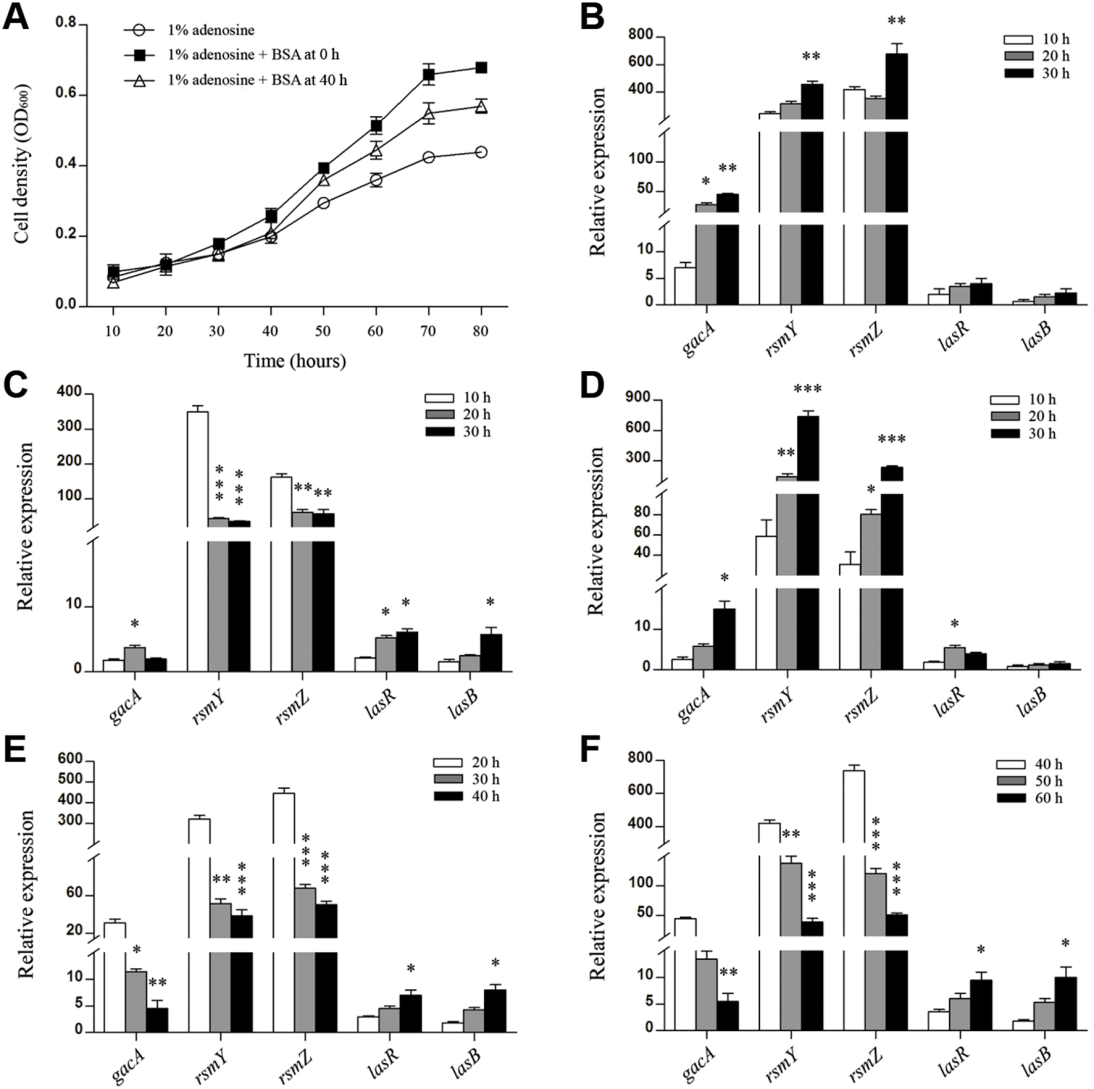
RsmYZ regulated cooperation was induced by environmental nutrient factors. (A) Growth of WT PAO1 in M9****minimal growth medium containing 1% adenosine (circle), 1% adenosine and 1% BSA as the dual carbon source (black square) and BSA was added after 40 hours cultivation (triangle). Expression of leadership and dependent genes of WT PAO1 were detected in M9****minimal growth medium containing (B) 1% adenosine; (C) 0.5% adenosine+0.5% BSA; (D) 0.5% +0.5% BSA and the supernatants were removed every 4 hours; (E) 1% adenosine and then 1% BSA was added after 20 hours cultivation; (F) 1% adenosine and then 1% BSA was added after 40 hours cultivation. Data are means ± SEM, and representative of three experiments. *, *P*<0.05; **, *P*<0.01; ***, *P*<0.001, one-way ANOVA (Tukey-Kramer post hoc).

Adenosine, a carbon source that can be degraded by LasR pathway-dependent intracellular private goods (degradative enzyme and nucleoside hydrolase) [27], and bovine serum albumin (BSA) that can only be used when it is broken down by the action of QS-dependent extracellular public good (elastase) [28], were designed to add into M9****minimal growth medium [1] to study the intracellular gene regulation of social cooperation. As shown in Fig. 3A, the growth rate of WT PAO1 was significant decreased when adenosine was used as the sole carbon source, however, the population expanded significantly when BSA was added into the culture. In contrast, leadership gene null mutants failed to grow in this medium (Fig. S3). These results support the hypothesis that the leadership-regulated activation of social cooperation may be nutrient-dependent. To probe the underlying mechanism of this regulation, the expression of major QS-related genes against various nutrient factors was then evaluated. As shown in Fig. 3B, when WT PAO1 was grown in M9-adenosine medium, the expression of small RNAs *rsmY* and *rsmZ* were significantly increased, and that the effect of *rsmY* and *rsmZ* on inducing *lasB* was profoundly dampened in the dual carbon source medium containing 0.5% adenosine and 0.5% BSA (Fig. 3C). To confirm these observations, WT PAO1 was cultured in a medium containing 0.5% adenosine and 0.5% BSA and removed the supernatants at 4****h intervals. Under these conditions, the expression of *rsmY* and *rsmZ* genes was continuously increased to a high level (Fig. 3D), strongly indicating their role in promoting population expansion. Additionally, the expression of *rsmY* and *rsmZ* genes decreased after BSA was added at later time points, respectively (Fig. 3E and F). Dandekar *et al.* [29] also found that QS can put a metabolic constraint on social cheating and incentivize the population to cooperate. This constraint should be due to the nutrient-dependent modulation of small RNAs, allowing quiescent bacteria to be vibrant in the appropriate environment. Therefore, our results here confirmed that the small regulatory RNAs function as efficient rheostats to coordinate cooperative behaviors by modulating individual excitability against various environmental factors. The production of private and public goods could be incentivized by nutrient-dependent bacterial metabolism, allowing quiescent bacteria to get activated in the appropriate environment.

### Environmental stresses could elevate the expression of *rsmY* and *rsmZ*

**Figure 4:**
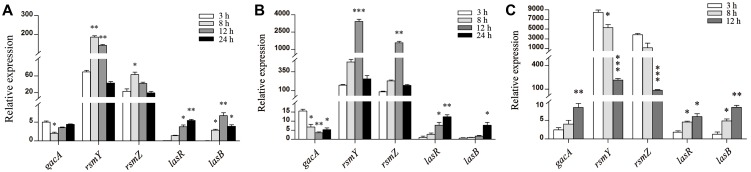
Expression of *rsmY* and *rsmZ* was dramatically increased under stressful circumstances. Expression levels of genes were detected in WT PAO1, which was cultivated in (A) LB broth medium (B) MH-S cells as well as (C) LB broth medium containing 2****µg/ml gentamicin at different time-points. Data are means ± SEM, and representative of three experiments. *, *P*<0.05; **, *P*<0.01, ***, *P*<0.001, one-way ANOVA (Tukey-Kramer post hoc).

**Figure 5:**
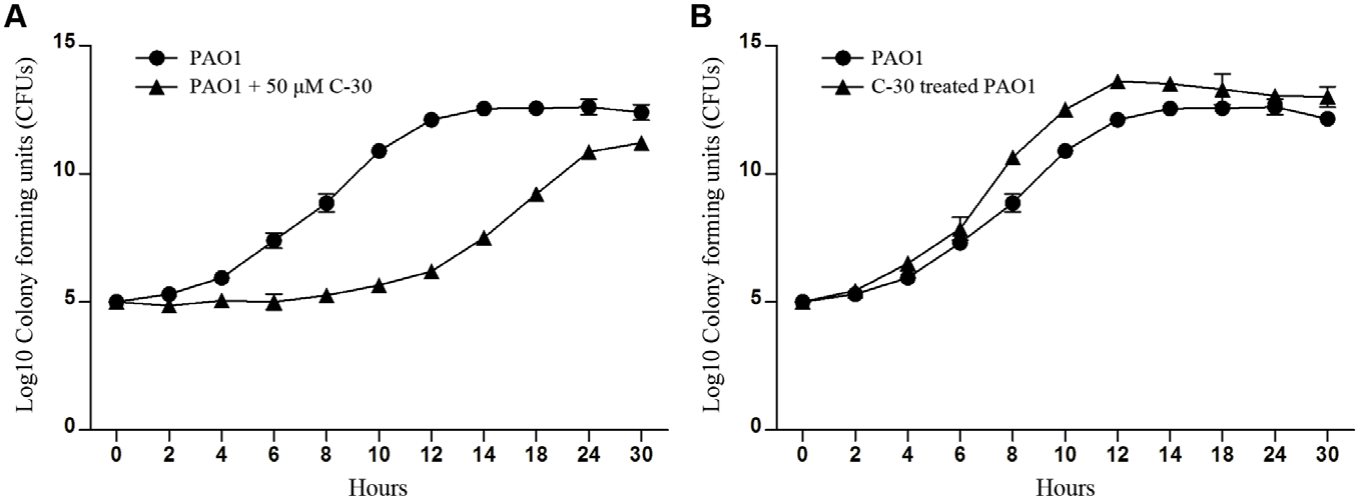
Furanone C-30-treated WT PAO1 showed enhanced cell proliferation compared with untreated group. (A) The growth of WT PAO1 was inhibited by furanone C before 12 hours culture. WT PAO1 was cultured with or without 50****µM furanone C-30 in LB broth medium and the CFUs were enumerated at designed time phases. (B) Furanone C-30-treated PAO1 cells harvested after 12 hour culture were immediately diluted to the same cell density (1.0×10^5^ ****CFU/ml) with untreated PAO1 for further cultivation. Data are means ± SEM, and representative of three experiments. Some error bars are too small to be seen.

**Figure 6:**
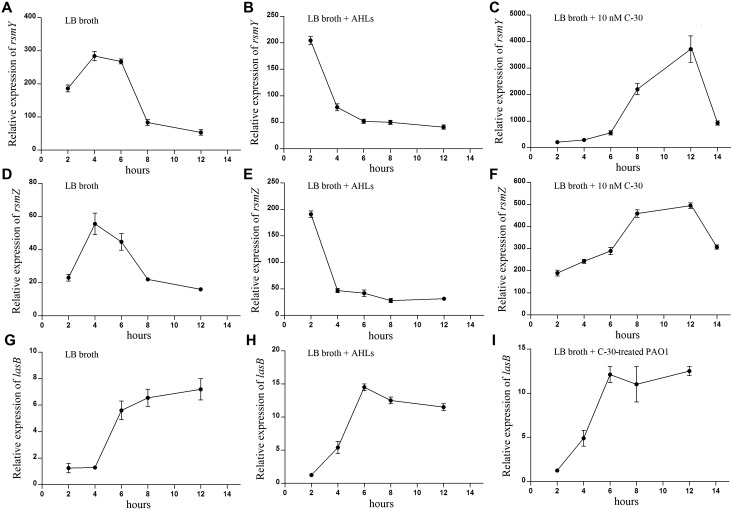
High levels of *rsmY* and *rsmZ* expression could cause an accelerated induction of cooperation. Time-dependent expression of *rsmY* and *rsmZ* of WT PAO1 was detected in LB broth (A and D), LB broth containing 20****µM 3O-C12-HSL and 20****µM C4-HSL (B and E), and LB broth containing 50****µM furanone C-30 (C and F). Time-dependent expression of *lasB* of same amounts WT PAO1 in LB broth (G) and LB broth containing 20****µM 3O-C12-HSL and 20****µM C4-HSL (H), and furanone C-30-treated PAO1 in LB broth (I).

**Figure 7:**
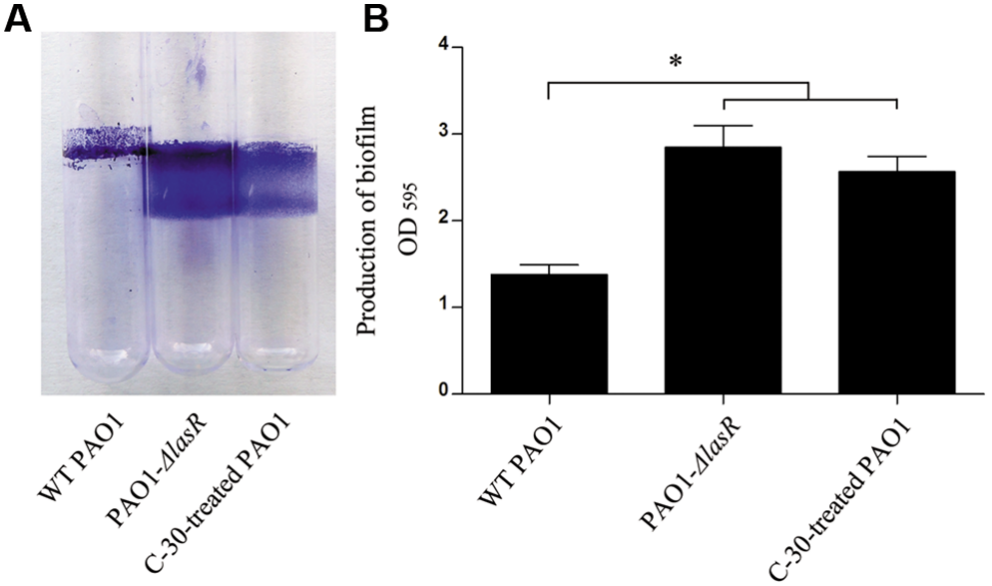
High levels of *rsmY* and *rsmZ* expression could cause an enhanced biofilm production after 24 h. Production of biofilm was detected by crystal violet staining (A) and quantified at OD_595_ (B). The data are represented as means ± SEM, six replicates per culture. *, *P*<0.05, one-way ANOVA (Tukey-Kramer post hoc).

We then set out to determine the environmental driver of leadership regulator for illustrating the social traits of bacteria by culturing WT PAO1 in different conditions. Compared with the control (Fig. 4A), the expression of *rsmY* and *rsmZ* genes continuously increased to a relatively high level and showed a delayed decrease when cultivated with mouse alveolar macrophage MH-S cells (to impose a harsh environment for the bacteria) (Fig. 4B). No significant increase in expression of *lasB* was detected until 24****h culture. The lack of activity may be due to the macrophage phagocytosis that constrains the autoinducer-triggered cooperation. Particularly, very high levels of *rsmY* and *rsmZ* were observed at the initial phase of culture containing gentamicin (2****µg/ml), and levels of these genes then decreased following the production of elastase (Fig. 4C). Additionally, the proliferation of WT PAO1 was significantly suppressed when co-cultured with 50****µM furanone C-30 in LB broth medium for 12****h, and then the growth rate began to increase indicating the fading effect of furanone C-30 (Fig. 5A). To further test this effect, the furanone C-30-treated WT PAO1 was collected at 12****h time point and cultured in LB broth medium. Interestingly, the furanone C-30-treated WT PAO1 showed an increased growth rate compared with the untreated PAO1 (Fig. 5B). Additionally, although the quiescent period of WT PAO1 was extended in the presence of furanone C-30 (Fig. 5A), *rsmY* and *rsmZ* transcripts accumulated to a high degree (Fig. 6A to F). In response to this intercellular simulating environment of abundant stress responders, the furanone C-30-treated PAO1 demonstrated enhanced growth rate and faster elastase production vs. the control (Fig. 5B and 6G to I). Moreover, the elevated expression of *rsmY* and *rsmZ* resulted in more biofilm formation than the control culture (Fig. 7). This result is consistent with previous study that the biofilm production of *gacA, rsmY* and *rsmZ* mutant strains were markedly impaired [8], [30]. Together, these results support the hypothesis that the variable effect of small RNA rheostats is beneficial for the bacterial community to gauge environmental stresses and become more intense to provide social defense to boost the population density.

## Discussion

This work provides experimental evidence that small regulatory RNAs act as early responders to efficiently modulate bacterial cooperation at low cell density. We found that: (*i*) bacterial social cooperation can be induced at a specific density in laboratory culture; (*ii*) the production of costly public goods is affected by the modulation of leadership gene expression upon environmental factors; and (*iii*) cooperative behaviors will be prematurely activated under the pressure of small regulatory RNAs when the bacterial population encounters environment threats.

Evolutionarily, bacteria are also social species that survive through mass communication [31]–[33]; thus a population at a low cell density without effective cooperation may succumb easily to environmental threats. Sandoz *et al.* [34] suggest that the expression of QS-controlled exoproduct-encoding genes was much higher at a low cell density under conditions that required these extracellular factors for growth. On the other hand, Dandekar and colleagues [29] found that bacterial private metabolism would constrain the emergence of cheaters who may dampen population fitness in low cell density. The current study provides clear support for the assumption that the leadership genes, especially small regulatory RNAs of *P. aeruginosa,* serve as early responders facilitating social cooperation. As a rheostat, these leadership genes also maintain the population in an ideal balance with improved collective defense and aggressive repertoires for feeding. RsmY and RsmZ primarily function during the cooperative resting phase by sensing environmental factors to modulate the private goods-dependent metabolism, and by determining whether cooperation is needed to prevent overproduction of costly public goods (Fig. 3), and the performances of these rheostats will be magnified against environmental stresses (Fig. 4 and 6). These results also offer a plausible explanation for why LasR sits atop a hierarchy of QS regulators and modulates the activity of RhlR and PqsR [35], [36], allowing individual cells to produce private goods for basal metabolism and for cooperation. Our results here should be seen as an empirical first step and shed new light on leadership-mediated homeostasis against environmental nutrient factors for social cooperation.

Importantly, since QS is critical for bacterial virulence, it has been considered as a promising target for novel therapeutics against chronic infections. Indeed, numerous studies have shown that halogenated furanones could impair the expression of QS-related genes and promote the clearance of *P. aeruginosa* from lungs of infected mice [23], [37]–[42]. However, our result here suggest that the suppressed QS can induce the accumulation of *rsmY* and *rsmZ,* and finally causing an accelerated expression of *lasB* and enhanced biofilm production with the fading effect (Fig. 6 and 7). It is predicted that there will be a fierce battle (from the host) if the bacteria are not completely cleared due to the enforcement of leadership genes. Thus, combination therapy of QS based-antivirulence drugs and antibiotics as an auxiliary method may help control *P. aeruginosa*-caused chronic lung infection.

Our data answer a critical but undervalued question concerning QS-controlled bacterial cooperative behaviors. QS provides a complicated but advantageous strategy to determine the initiation and progression of social cooperation. The induction of cooperative behavior is nutrient-dependent, and QS restrains over-production of costly public goods if the surrounding environment of the bacteria does not call for the corresponding extracellular factors. The cooperative behaviors are tightly regulated and can be inappropriately activated when facing an alarming threat to the population fitness. Importantly, we discover that these processes involve the variation of small regulatory RNAs expression, which function as efficient rheostats in immediate response to environmental stresses to quickly synthesize both private and public goods. We believe that further mechanistic studies will greatly impact the current understanding of sociomicrobiology and pathogenesis mechanisms and provide new insight into more effective strategies to control bacterial infection.

## Supporting Information

Figure S1 **Colony forming units of different** ***P. aeruginosa*** **mutants in LB broth medium.** All the data are represented as means ± SEM, three replicates per culture. *, *P*<0.001, one-way ANOVA (Tukey-Kramer post hoc).
(TIF)

Figure S2 **Time-dependent expression of QS-related genes of different** ***P. aeruginosa*** **mutants in LB broth medium.** Relative expression of main QS related genes in *gacA* mutant (A), *rsmY* mutant (B), *rsmY* mutant (C), *rsmY* and *rsmZ* double mutant (D), and *rsmA* mutant (E). All the data are represented as means ± SEM, three replicates per culture. *, *P*<0.05, **, *P*<0.01, one-way ANOVA (Tukey-Kramer post hoc).
(TIF)

Figure S3 **Colony forming units of different** ***P. aeruginosa*** **mutants in M9 minimal growth medium containing 1% adenosine as the sole carbon source.** All the data are represented as means ± SEM, three replicates per culture. *, *P*<0.001, one-way ANOVA (Tukey-Kramer post hoc).
(TIF)

Table S1 **Specific RT-PCR primers used in this study.**
(DOCX)

Table S2 **Spearman’s correlation between the expression levels of different QS related genes during the growth of WT PAO1.**
(DOCX)
