# Supplementary figures and images for: Genes as Early Responders Regulate Quorum-Sensing and Control Bacterial Cooperation in Pseudomonas aeruginosa
Source: PLoS One. 2014 Jul 9;9(7):e101887. doi: 10.1371/journal.pone.0101887 (PMC4090235; doi:10.1371/journal.pone.0101887)

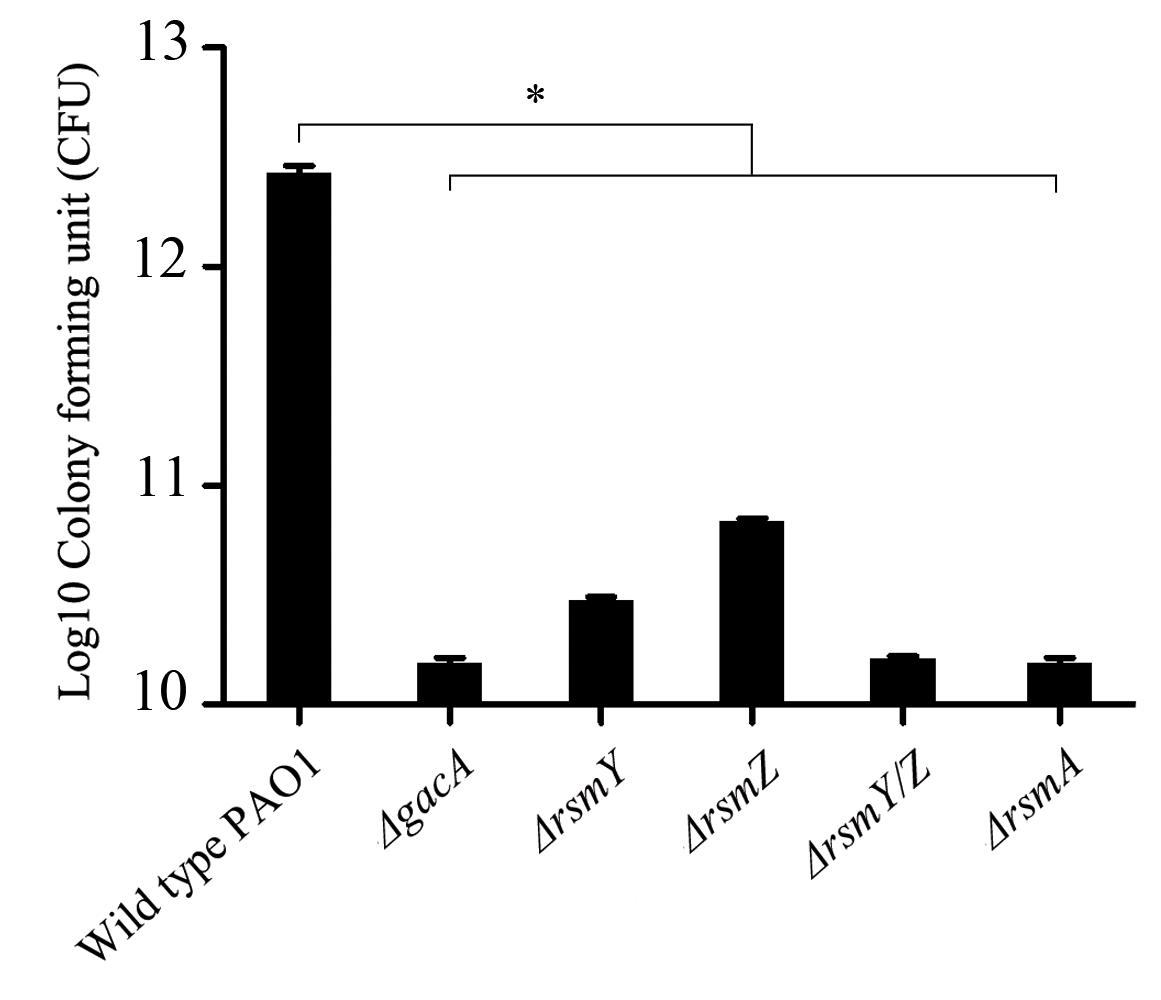

Supplement: Figure S1 — Colony forming units of different P. aeruginosa mutants in LB broth medium. All the data are represented as means ± SEM, three replicates per culture. *, P<0.001, one-way ANOVA (Tukey-Kramer post hoc). (TIF) [file pone.0101887.s001.tif]

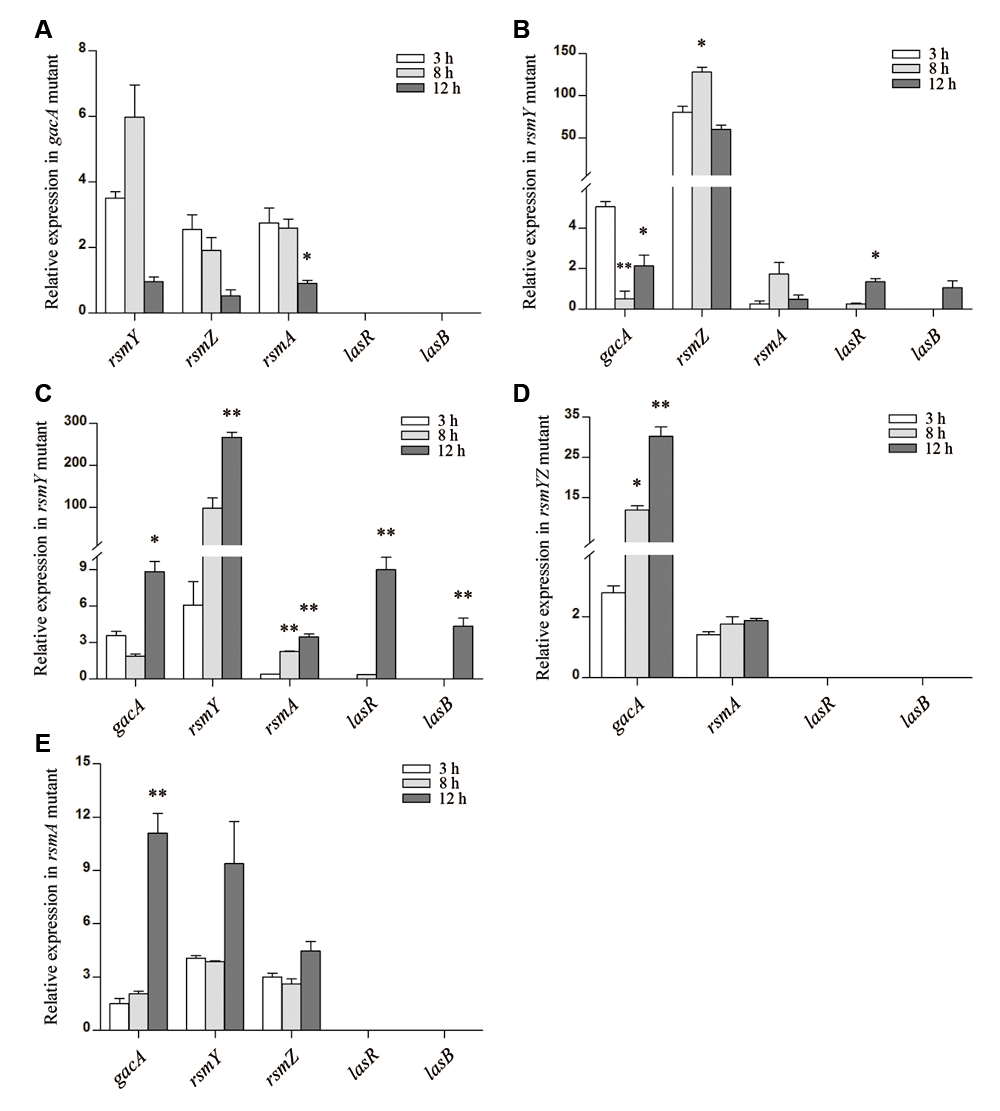

Supplement: Figure S2 — Time-dependent expression of QS-related genes of different P. aeruginosa mutants in LB broth medium. Relative expression of main QS related genes in gacA mutant (A), rsmY mutant (B), rsmY mutant (C), rsmY and rsmZ double mutant (D), and rsmA mutant (E). All the data are represented as means ± SEM, three replicates per culture. *, P<0.05, **, P<0.01, one-way ANOVA (Tukey-Kramer post hoc). (TIF) [file pone.0101887.s002.tif]

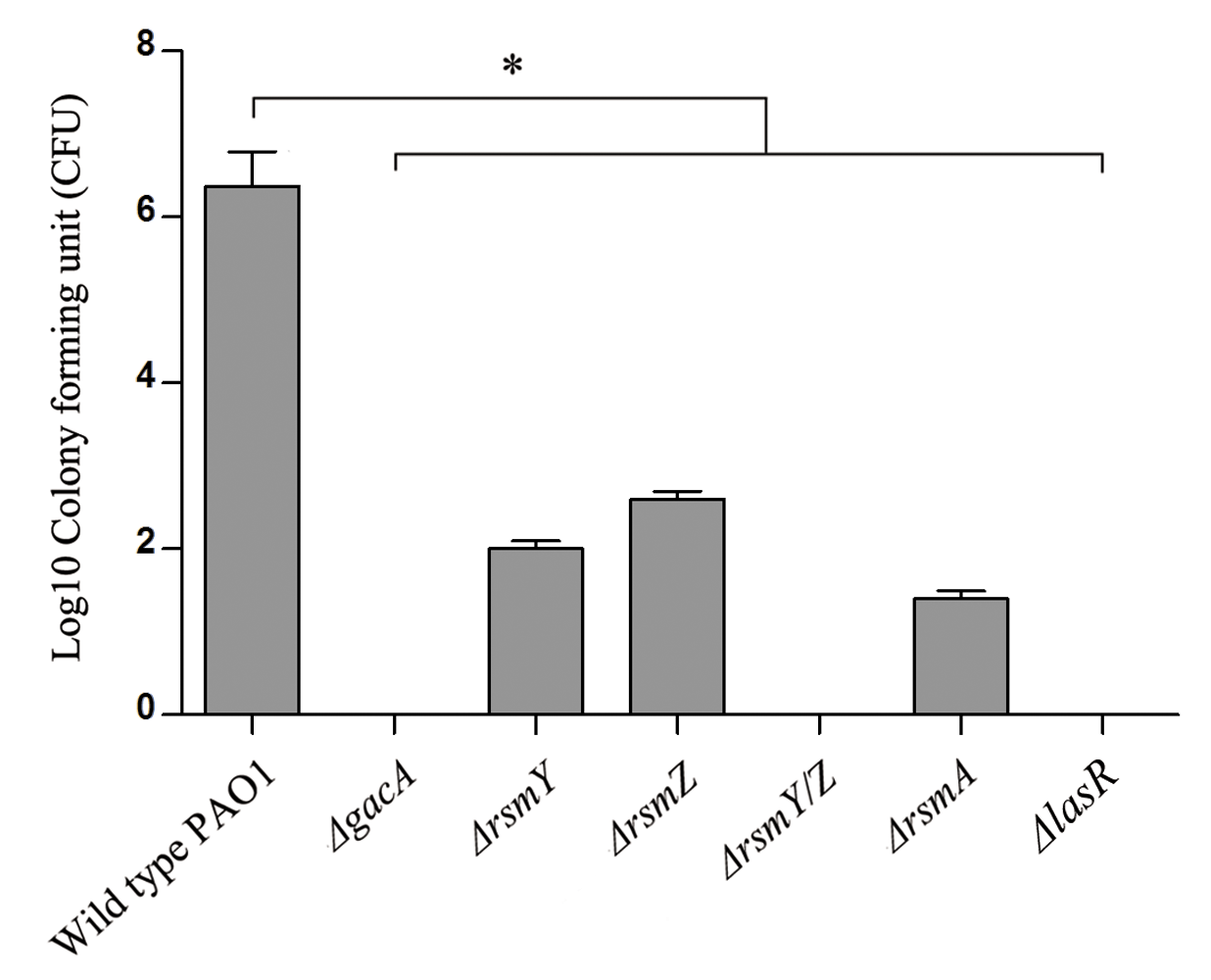

Supplement: Figure S3 — Colony forming units of different P. aeruginosa mutants in M9 minimal growth medium containing 1% adenosine as the sole carbon source. All the data are represented as means ± SEM, three replicates per culture. *, P<0.001, one-way ANOVA (Tukey-Kramer post hoc). (TIF) [file pone.0101887.s003.tif]
