# Supplementary material for: Genes as Early Responders Regulate Quorum-Sensing and Control Bacterial Cooperation in Pseudomonas aeruginosa
Source: PLoS One. 2014 Jul 9;9(7):e101887. doi: 10.1371/journal.pone.0101887 (PMC4090235; doi:10.1371/journal.pone.0101887)
Supplement: Table S1 — Specific RT-PCR primers used in this study. (DOCX) [file pone.0101887.s004.docx]

**Table S1. Specific RT-PCR primers used in this study.**

| Gene assayed | Primer sequence |
| --- | --- |
| *gacA* | Forward: 5’- CCGACTGCGGTGAAGACTGT -3’ |
|  | Reverse: 5’- GGTGACTACCACGACCTTGATG -3’ |
| *gacS* | Forward: 5’- CAGCAGGACTACCTCACGAC -3’ |
|  | Reverse: 5’- AGGTCGCGGAGATTGAAAGG -3’ |
| *rsmA* | Forward: 5’- TACTGGGTGTCAAAGGGAAC -3’ |
|  | Reverse: 5’- ATGGTTTGGCTCTTGATCTT -3’ |
| *rsmY* | Forward: 5’- GCCAAAGACAATACGGAAAC -3’ |
|  | Reverse: 5’- TCTATCCTGACATCCGTGCT-3’ |
| *rsmZ* | Forward: 5’- TACAGGGAACACGCAACC -3’ |
|  | Reverse: 5’- CCACTCTTCAGTCCCTCGT -3’ |
| *lasR* | Forward: 5’- CTTCATCGTCGGCAACTAC -3’ |
|  | Reverse: 5’- GTCTGGTAGATGGACGGTTC -3’ |
| *lasI* | Forward: 5’- TGCGTGCTCAAGTGTTCAAGG -3’ |
|  | Reverse: 5’- TGTCCAGAGTTGATGGCGAAA -3’ |
| *rhlR* | Forward: 5’- GCTCCTCGGAAATGGTGGT -3’ |
|  | Reverse: 5’- GGAAAGCACGCTGAGCAAAT -3’ |
| *rhlI* | Forward: 5’- TCCGCAAACCCGCTACATC -3’ |
|  | Reverse: 5’- TCTCGCCCTTGACCTTCTGC -3’ |
| *rpoS* | Forward: 5’- GCCTGAACGAACGGGTGACT -3’ |
|  | Reverse: 5’- CACCTCACGCTGCTTGTCG -3’ |
| *exsA* | Forward: 5’- GGTAAACAAGGAAGAGGGCGTAT -3’ |
|  | Reverse: 5’- GGACGAAGCCTTGTAGAAACTGG -3’ |
| *lasB* | Forward: 5’- ATCGGCTACGACATCAAGAAGG -3’ |
|  | Reverse: 5’- CCGCTGTTGTAGTTGCTGGTG -3’ |
| *rpoA* | Forward: 5’- TCGCATCCTGTTGTCCTCCA -3’ |
|  | Reverse: 5’- TTAGCCAGGGTCAGCGTCA -3’ |
