# Supplementary material for: Genes as Early Responders Regulate Quorum-Sensing and Control Bacterial Cooperation in Pseudomonas aeruginosa
Source: PLoS One. 2014 Jul 9;9(7):e101887. doi: 10.1371/journal.pone.0101887 (PMC4090235; doi:10.1371/journal.pone.0101887)
Supplement: Table S2 — Spearman’s correlation between the expression levels of different QS related genes during the growth of WT PAO1. (DOCX) [file pone.0101887.s005.docx]

**Table S2. Spearman’s correlation between the expression levels of different QS related genes during the growth of WT PAO1.**

|  | *gacA* | *gacS* | *rsmA* | *rsmY* | *rsmZ* | *lasR* | *lasI* | *rhlR* | *rhlI* | *rpoS* | *exsA* | *lasB* |
| --- | --- | --- | --- | --- | --- | --- | --- | --- | --- | --- | --- | --- |
| *gacA* |  |  |  | － | － | － | － |  |  | － | － |  |
| *gacS* |  |  | － |  |  |  |  |  |  |  |  | － |
| *rsmA* | － |  |  |  |  |  |  |  |  |  |  | ＋ |
| *rsmY* | ＋ |  |  | ＋ |  | ＋ |  |  | ＋ | ＋ | ＋ |  |
| *rsmZ* | ＋ |  |  |  |  | ＋ |  |  | ＋ | ＋ | ＋ |  |
| *lasR* | － |  |  | ＋ | ＋ |  |  |  | ＋ | ＋ | ＋ |  |
| *lasI* |  |  |  |  |  |  |  | ＋ |  |  |  |  |
| *rhlR* |  |  |  |  |  |  | ＋ |  |  |  |  |  |
| *rhlI* | － |  |  | ＋ | ＋ | ＋ |  |  |  | ＋ | ＋ |  |
| *rpoS* | － |  |  | ＋ | ＋ | ＋ |  |  | ＋ |  | ＋ |  |
| *exsA* | － |  |  | ＋ | ＋ | ＋ |  |  | ＋ | ＋ |  |  |
| *lasB* | － |  | ＋ |  |  |  |  |  |  |  |  |  |

＋, positive correlation; －, negative correlation; blank, not significant correlation.

*P* value of < 0.01 was considered statistically significant.
